# Frequent loss of heterozygosity and altered expression of the candidate tumor suppressor gene '*FAT' *in human astrocytic tumors

**DOI:** 10.1186/1471-2407-9-5

**Published:** 2009-01-07

**Authors:** Kunzang Chosdol, Anjan Misra, Sachin Puri, Tapasya Srivastava, Parthaprasad Chattopadhyay, Chitra Sarkar, Ashok K Mahapatra, Subrata Sinha

**Affiliations:** 1 Department of Biochemistry, All India Institute of Medical Sciences, New Delhi, India; 2 Pathology, All India Institute of Medical Sciences, New Delhi, India; 3 Neurosurgery, All India Institute of Medical Sciences, New Delhi, India; 4 Barrow Neurological Institute, St. Joseph's Hospital & Medical Center, Phoenix, AZ, USA; 5 Laboratory of Molecular & Tumor Immunology, Earle A Chiles Research Institute, Providence Portland Medical Center, 4805 NE Glisan Street Portland, OR, USA; 6 Sanjay Gandhi Postgraduate Institute of Medical Sciences (SGPGI), Raebareli Road Lucknow, India

## Abstract

**Background:**

We had earlier used the comparison of RAPD (Random Amplification of Polymorphic DNA) DNA fingerprinting profiles of tumor and corresponding normal DNA to identify genetic alterations in primary human glial tumors. This has the advantage that DNA fingerprinting identifies the genetic alterations in a manner not biased for locus.

**Methods:**

In this study we used RAPD-PCR to identify novel genomic alterations in the astrocytic tumors of WHO grade II (Low Grade Diffuse Astrocytoma) and WHO Grade IV (Glioblastoma Multiforme). Loss of heterozygosity (LOH) of the altered region was studied by microsatellite and Single Nucleotide Polymorphism (SNP) markers. Expression study of the gene identified at the altered locus was done by semi-quantitative reverse-transcriptase-PCR (RT-PCR).

**Results:**

Bands consistently altered in the RAPD profile of tumor DNA in a significant proportion of tumors were identified. One such 500 bp band, that was absent in the RAPD profile of 33% (4/12) of the grade II astrocytic tumors, was selected for further study. Its sequence corresponded with a region of *FAT*, a putative tumor suppressor gene initially identified in *Drosophila*. Fifty percent of a set of 40 tumors, both grade II and IV, were shown to have Loss of Heterozygosity (LOH) at this locus by microsatellite (intragenic) and by SNP markers. Semi-quantitative RT-PCR showed low *FAT *mRNA levels in a major subset of tumors.

**Conclusion:**

These results point to a role of the *FAT *in astrocytic tumorigenesis and demonstrate the use of RAPD analysis in identifying specific alterations in astrocytic tumors.

## Background

Astrocytic tumors are the most frequent human gliomas; they are the second most common cause of cancer mortality in young adults after leukemia. WHO has classified astrocytic tumors into 4 grades [1]. Grade I (Pilocytic Astrocytomas) have a benign outcome and have genetic pathways that differ from the three higher grades. However Grades II to IV follow the same lineage of progression and Grade II (Low-grade Diffuse Astrocytomas, DA) have an inherent tendency to progress to grade III (Anaplastic Astrocytoma, AA) and grade IV (Glioblastoma Multiforme, GBM)[2,3]. Molecular alterations associated with these histopathological classes have also been identified and studied extensively. The median survival time of Grade II and IV tumors is 6 years and 1 year respectively [4], and over the years, not much has changed regarding their outcome. Malignant transformation of glial cells is a complex process[5] that is still incompletely understood [6,7].

We had earlier used RAPD-DNA fingerprinting techniques [8] to identify alterations in tumor DNA in a manner not selected for locus [9,10]. While RAPD primers define distinct loci, their selection is random and not locus based. We have also used this technique to measure the extent of intra-tumor genetic heterogeneity [11,12] and genomic instability in high and low grade tumors [13] and the role of repeat sequences in the generation of instability [14,15]. This technique scans the whole genome for complimentarity at any locus and thus enhances the chances of detecting novel altered genomic regions in tumors. Furthermore, RAPD screening can be performed on a small amount of tumor DNA (50–100 ng of DNA per PCR). This is important because of the possibility of shearing and low retrieval after extracting tumor DNA from cryostat sections. In most of our earlier studies we concentrated on genomic instability i.e. the RAPD changes, which varied from tumor to tumor. In this study, we have used the RAPD-PCR technique to identify and characterize novel alterations in the human astrocytic tumors of WHO grade II (DA) and grade IV (GBM) using normal leucocyte DNA of the same patient as control. The altered bands (gained, amplified or lost) in the tumor RAPD profiles are used to document, quantify and characterize the nature of alterations. We focused on the alterations that were common to a significant proportion (more than 25%) of the astrocytic tumors studied and characterized one of them. This was done in order to identify genomic changes that may be significant to the tumorigenic process.

We initially used five different primers to compare the RAPD profile of 23 astrocytic tumors (12 grade II and 11 grade IV) with their corresponding normal leucocyte DNA. One altered band, which was absent in the RAPD profile of 33% of the Grade II tumors studied, was found to be homologous to the tumor suppressor gene '*FAT' *on chromosome 4q34-35. Heterozygosity analysis performed in a larger set of 40 tumors and gene expression studies [by semi quantitative reverse transcriptase (RT)-PCR] indicate that the *FAT *may be involved as a tumor suppressor gene in primary human glioma.

## Methods

The work has been approved by the ethics committee of our institute. Samples were collected with prior informed consent from each patient.

### Samples processing and DNA extraction

Samples of WHO grade II and IV human astrocytic tumors were obtained from patients who were undergoing surgery at Neurosurgery Operation Theatre of All India Institute of Medical Sciences (AIIMS), India. The number of Grade III tumors we get from our clinical collaborators are much fewer than Grade II and Grade IV. Hence we chose grade II as an example of Low Grade Astrocytoma and Grade IV as an example of High Grade Astrocytoma. Tissue samples were immediately stored frozen at -70°C. Cryosectioning was done and only those sections were taken which showed more than or equal to 80% neoplastic cells. Leucocyte DNA of the same patient was used as a control. DNA extraction and quantification was done as described by us previously[11].

### RAPD-PCR analysis and scoring of alterations

RAPD-PCR was done with 5 RAPD 10 mer primers, purchased from Genosis, USA (Table 1). PCR was performed with the precautions described by us earlier[14] in PTC200 thermal cycler (MJ Research, USA). Products were resolved in a 1.5% agarose gel, stained with ethidium bromide, visualized on a UV transilluminator and documented. Scoring of the alteration(s) was done by comparing the RAPD profile of the normal and tumor DNA. The alteration(s) observed were confirmed by three independent experiments and scoring done by 2 independent observers (KC and AM). Twenty three astrocytic tumors (12 grade II and 11 grade IV) were studied by this technique.

**Table 1:** Details of the RAPD primers, Microsatellite markers and SNP markers used.

RAPD Primers					
**Serial no**.	**RAPD Primer ID**	**Sequence (5'-3')**	**GC content (%age)**	**Annealing temp**.	**MgCl_2 _(mM)**

**1**.	**80/07**	**GCACGCCGGA**	80	38°C	3.0

**2**.	**80/01**	**GCACCCGACG**	80	35°C	3.0

**3**.	**70/04**	**CGCATTCCGC**	80	35°C	3.0

**4**.	**70/01**	**CATCCCGAAC**	70	38°C	3.0

**5**.	**60/31**	**TGCGCGATCG**	70	38°C	3.0

**Microsatellite markers**					

**Serial no**.	**Microsatellite marker ID**	**Primer sequences (5'-3')**		**Annealing temp**.	**Product size (bp)**

**1**.	**D4S3173**	**Left Primer: TTGGAGTCCCTGAAGGACC** **Right primer: TACAAACACCAAGCCCCTTC**		57°C	103–104

**2**.	**D4S2827**	**Left Primer: GACGGGAGAGAAAATGCATT** **Right primer: TCAATAAGCACCAAAACTACTCAG**		55°C	132–133

**3**.	**D4S1295**	**Left Primer:TAACAAAACCATCTCCAAACTTCTC** **Right primer: GTGGACAGTACAACACAGTTGAC**		57°C	170

**4**.	**D4S2643**	**Left Primer:TTGTCCAATAACTCTTTTCCTAACA** **Right primer: CTGATTCCTATATCAATCTTGGCC**		57°C	179–180

**5**.	**D4S2672**	**Left Primer: TAAAGATGTGATTTGTATTGCATTG** **Right primer: AGTTTCAGCCTCTCAAAATTTCA**		57°C	199–200

**SNP markers**					

**Serial no**.	**SNP Cluster ID**	**Primer sequences (5'-3')**	**Annealing temp**.	**Product size (bp)**	**Restriction endonucleases**

**1**.	**rs2276930**	Left primer: GCCGTAACTAACCTCGGCATC Right primer: GCCTCACGCTCCCCGAGCGCA	61°C	270	BstN1

**2**.	**rs3733414**	Left primer: CGCCCCAGTTCTCTTCTGT Right primer: TGTTGTTACTTCAAGTTCAAAATGG	60.8°C	290	Rsa1

**3**.	**rs455600**	Left primer: TCATTGACTTTTGCTTTTCCTAA Right primer: CCAACGAATCAGCAAAGAAC	60.8°C	299	Acc1

**4**.	**rs213090**	Left primer: GGCAGTTGAAATTTACTTTATAGTTCT Right primer: AGACTGCAGTGTGCAATTCTG	61°C	394	HaeIII

**5**.	**rs450320**	Left primer: CGAAATCCTACTCCTGGCTTT Right primer: GCATCACTCCTGCTCCTCAT	60.8°C	349	Hpy1881

**6**.	**rs7663350**	Left primer: TGGTTTTGGTGAAGCATAAGAA Right primer: AAGTTATCCAGGATTTCAATCTCA	60.8°C	400	Sau3A1

**7**.	**rs1193110**	Left primer: AGACCCTTTCAGCCAGTTCA Right primer: AGTGAGATGAGGCCAGTGCT	61°C	369	Msp1

**8**.	**rs1298865**	Left primer: TTGTCATCCTTCCTTTTAGCAA Right primer: GATCCTTCAATGAATATGTTCTTCTC	60.8°C	400	Dde1

### Characterization of altered fragment

The RAPD gel of the samples (paired tumor and control DNA) showing an altered band profile in tumors was transferred to a nylon membrane for Southern analysis[14]. The altered fragment from one of the normal sample from a separate gel was excised and eluted from the gel using Qia quick gel extraction kit (Qiagen, USA). The eluted band was PCR radiolabelled and used as probe for Southern hybridization of a gel containing paired tumor and control DNA PCR products showing alteration. The eluted band was then cloned in a pGEMT-Easy vector system (Promega, USA). The insert was again confirmed by Southern hybridization by using the gel eluted radiolabeled fragment as probe. Sequencing of the cloned fragment was done by using Thermosequenase kit (Amersham, USA) with fluorescent labeled dideoxy nucleotides on an ABI-Prism automated DNA sequencer (Appiled Biosystem, USA). Homology search of the sequences was done using the BLAST search tool with the human genome sequences database.

### Characterization of the RAPD primer binding sites

In order to identify if changes had occurred in the RAPD primer binding sites, a specific primer pair (forward 5' ACCCgTgTTTTCCAgCTTT 3' and reverse 5' CTCTgCCTCTCgACCAAAAC 3') was designed to amplify the FAT region corresponding to the altered RAPD band identified including about 100 bases on either side flanking the altered band. The PCR using specific primer pair was done directly from genomic DNA from two sample pairs (tumor and corresponding normal DNA) shown to have alteration in the tumors. The PCR product was resolved in 1.5% agarose gel, stained with Ethidium bromide and documented. The PCR product was eluted from all the samples (two normal and two tumors) separately from the gel using Qia quick gel extraction kit (Qiagen, USA) and sequenced. Homology search of the sequences was done by BLAST 2 search tool with the *FAT *sequence to look for alterations in the RAPD primer binding regions.

### Loss of heterozygosity (LOH) studies

LOH analysis of the *FAT *locus was done using five microsatellite markers and ten SNP markers on 40 human astrocytic tumors (20 grade II and 20 grade IV). One microsatellite marker used was intragenic to the *FAT *gene and four flanking the gene. All the SNP markers used were intragenic to the *FAT *gene.

#### Microsatellite Markers: Selection and analysis

Microsatellite markers (one intragenic and two each flanking on both 5' and 3' side of the *FAT *gene, within 0.2 Mb of the gene) were selected. The markers studied were D4S3173, D4S2827, D4S1295, D4S2643 and D4S2672. The sequences of the forward and reverse primers of the markers were taken from the NCBI UNISTAT database and primers were obtained from Research Genetics, Inc, USA (Table 1).

The PCR cycling conditions and the annealing temperature were standardized. The PCR product was resolved onto a 6% denaturing gel containing 7 M Urea cast on the BioRad midigel apparatus. After the electrophoresis for visualization and analysis of the bands the gel was stained with 10 mg% ethidium bromide[16], visualized on UV transilluminator and documented in Gel Documentation System (Alpha Innotech Corporation, USA). Each experiment was repeated twice and scoring done by 2 independent observers (KC and TS).

For heterozygosity analysis the microsatellite pattern of tumor DNA was compared to its corresponding leucocyte DNA. Only informative (heterozygous) samples were considered for LOH analysis. LOH observed with even one of the marker in the tumor was taken as LOH in the tumor at the locus.

#### SNP markers: Selection and analysis

Genotypic and allelic frequency of 365 SNPs present in *FAT *gene was obtained from Ensembl database. SNPs reported to have a high heterozygosity status were chosen. Hundred SNPs with high heterozygosity status were checked for the presence of restriction endonuclease site using DNA STAR program. We found 30 SNPs with a restriction site, of which only 10 SNPs were found to have had a unique restriction site within the polymorphic region when flanking 400–500 bps. Primers were designed for each SNP so as to obtain a PCR product of 300–400 bp, which on restriction digestion gave bands of unequal size. All the primers were commercially synthesized from Integrated DNA Technologies, Inc. (Table 1). The PCR products were purified by chloroform treatment followed by ethanol precipitation. The digestion of the PCR products was done with the appropriate restriction enzyme and resolved on 2% agarose gel, stained with ethidium bromide and visualized on UV transilluminator and documented.

For heterozygosity analysis the SNP-PCR-RFLP pattern of tumor DNA was compared to its corresponding leucocyte DNA. For LOH analysis, only those sample pair were considered in which the normal DNA had shown a heterozygous pattern. For scoring changes, LOH with even one SNP marker was considered to be indicative of LOH of the locus for a particular sample. LOH frequency for each SNP was calculated separately as percentage of all informative samples for that particular SNP.

### RNA extraction and Reverse transcription- PCR analysis

RNA extraction from tumor tissues was done[17]. Reverse transcriptase (RT)-PCR was done using an RNA PCR kit (Perkin Elmer Corporation, USA) following the manufacturer's instructions. PCR of the reverse transcribed product was done using gene (*FAT *gene) specific primer pair (Forward 5' TTCAAAATAggTgAAgAgACAggTg 3' & reverse 5' TTgTgATgAgACCTgTTTTAggATg 3') in PTC 200 thermal cycler (MJ Research). Expression level of glyceraldehyde-3-phosphate dehydrogenase (GAPDH) was used as an internal control. The primer pair used for GAPDH amplification was; Forward 5' CCAAggTCATCCATgACAACTTTggT 3' & reverse 5' TgTTgAAgTCAgAggAgACCACCTg 3'. Total of 18 astrocytic tumors (9 grade II and 9 grade IV) and 2 cell-lines (U87MG & U373MG) were subjected to RT-PCR analysis. Because of non-availability of suitable samples, these were different from the tumors used for the LOH studies. The amplification products were resolved in 2% agarose gel, documented in Gel Documentation System (Alpha Innotech Corporation, USA) and analyzed by densitometry (using ChemiImager Software) for Integrated Density Values (IDV). The densitometric value of the GAPDH band was used to normalize the *FAT *band. On the basis of IDV, tumors were divided into three groups (with IDV of <0.75, 0.75–1.5 & >1.5). The mean and standard deviation (SD) of the integrated density values (IDV) of the samples in these 3 groups was calculated and the means compared to analyze and represent the expression results.

## Results

### RAPD-fingerprinting of human astrocytic tumors and characterization

**Figure 1:**
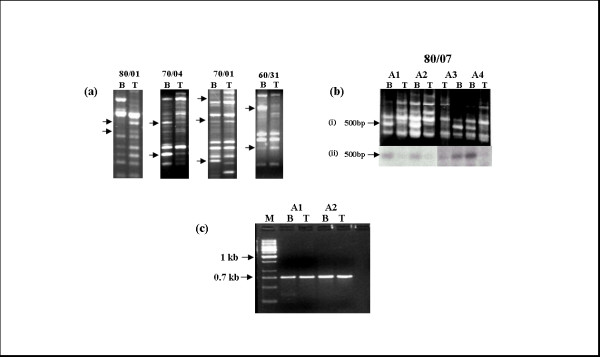
**(a) Representative RAPD gel profile showing alterations (arrows) in the form of loss/gain/change in intensity of band(s) in tumor (T) as compared to normal leucocyte DNA (B) of the same patient, with different primers (primer nos. 80/01, 70/04, 70/01, 60/31)**. (b-i) RAPD gel profile of primer no. 80/07 indicating the frequent loss of a 500 bp band (arrow) in astrocytic tumors (T) as compared to the corresponding normal leucocyte DNA (B). (b-ii) Southern blot of the same RAPD profile, Southern hybridization was done to confirm the altered fragment with a radiolabeled probe prepared from 500 bp altered band eluted from the RAPD profile of normal DNA from another gel. (c) Amplification of normal (B) and tumor (T) DNA with loss of 500 bp band with specific primer pair designed to amplify the FAT gene corresponding to the altered band along with 100 bp on either side. Bands were eluted from the gel and sequenced to look for deletion(s)/mutation(s) at RAPD primer binding sites. M represents molecular marker.

RAPD-PCR was done on 23 astrocytic tumors (12 Grade II and 11 Grade IV) along with the corresponding normal leucocyte DNA with 5 RAPD primers mentioned (Table 1). The alterations in the tumors were detected in the form of loss/gain and/or change in the intensity of band. Most of these alterations were unique to a particular sample and were not consistent from tumor to tumor (Fig 1a). With primer no. 80/07 loss of a band of ~500 bp was detected in 33% (4/12) of the grade II tumors (Fig 1b–i). We named the altered fragment – 80/07/A2 (80/07 is the primer number; A2 is the sample number from which it was first identified). Confirmation of consistent change by Southern blotting with PCR-radiolabeled 80/07/A2 fragment showed that the altered fragment was unique in the RAPD pattern in various samples mentioned (Fig 1b–ii). The loss observed in the Southern blotting, corresponded with the agarose gel profile. This further confirmed that the eluted band was similar to the band lost in the tumors. It was then cloned and the identity of the clone was further confirmed by Southern hybridization and sequencing. BLAST search of the sequence (Gen Bank Accesion no. AF250763) showed 100% homology to *FAT*, a putative tumor suppressor, at exon2-intron2 junction on chromosome 4q34-q35 locus. *FAT *is homologue of the Drosophila tumor suppressor gene *fat *and is a member of the Cadherin gene family. It is a transmembrane protein of nearly 4600 amino acid residue with 34 tandem cadherin repeats, 5 EGF like repeats and a lamin A-G domain.

### Characterization of the RAPD primer binding sites

Two sample pairs, showing loss of band in the tumors, when subjected to PCR using specific primer pair, a PCR product of ~700 bp was obtained in all the samples including both normal leukocyte and tumors (Fig 1c). The PCR product of all the samples (normal and tumors) was eluted, sequenced and searched for mutations or deletions at the RAPD primer (80/07) binding regions (both 3' and 5') by performing BLAST 2 search with the *FAT *gene sequence. No mutations or deletions were detected at the primer binding regions of all the four samples (two normal and two tumors) analyzed. Hence the reason for the altered RAPD profile consistently and specifically at this band in a high percentage of tumors could not be ascertained. This however led us to investigate the region by other methods, including LOH studies.

### LOH analysis of the *FAT *locus

**Figure 2:**
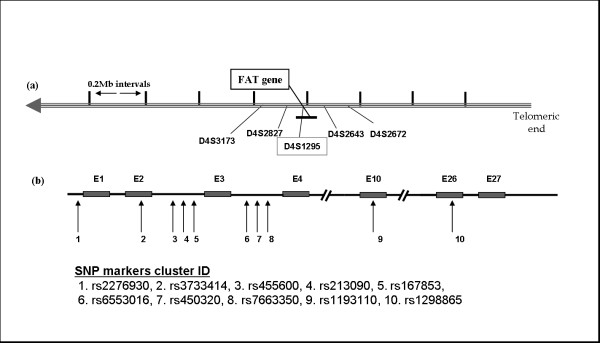
**Schematic representation of the marker loci used for LOH analysis**. (a) chromosome 4q35 loci with the microsatellite markers from within and near to the *FAT *gene}. (b) Location of SNP markers in relation to the exons and introns of *FAT *gene. E indicates exon, numbered arrows indicate the SNP markers selected for LOH study. The Cluster id of SNP markers selected and used are mentioned.

**Figure 3:**
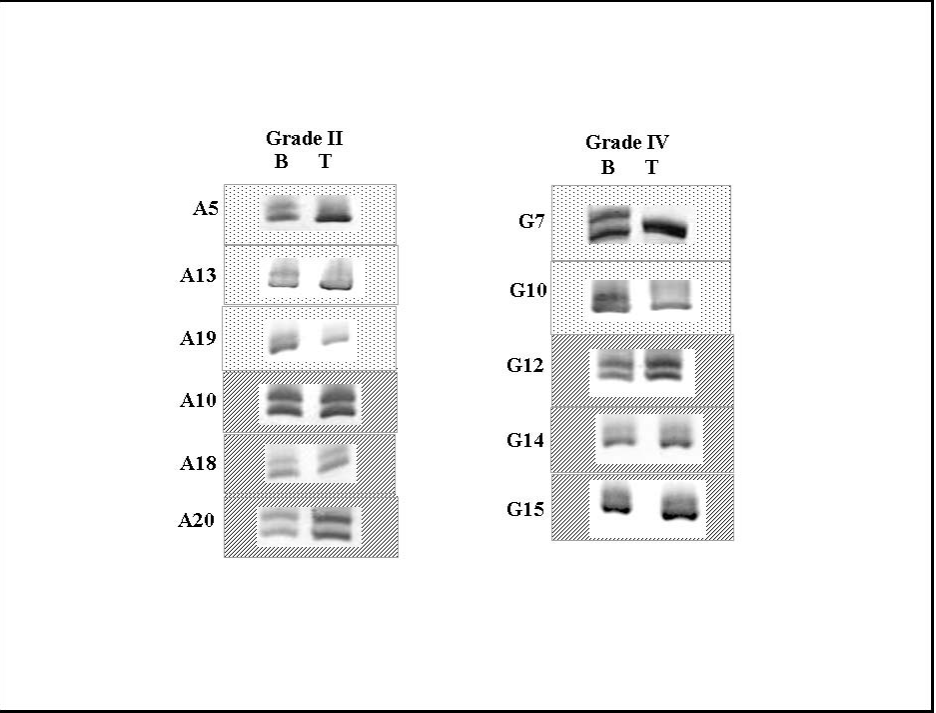
**Heterozygosity status in WHO grade II and IV astrocytic tumors with D4S1295 (intragenic to *FAT*) marker**. Heterozygous samples are shown in boxes with line patterns and samples showing LOH are in dotted boxes. B indicates constitutive normal leucocyte DNA and T indicates tumor DNA. Out of 5 microsatellite markers used, only one marker (D4S1295) was informative and rest, all were uninformative (not shown in the figure). All photographs have been cropped and inverted to a white background for clarity.

The microsatellite and SNP markers used for LOH analysis are schematically represented in relation to the *FAT *gene in Fig 2 and the primer sequences are shown in table 1. Of the five microsatellite markers used, only the intragenic marker i.e. D4S1295 was informative, while the other four markers were uninformative. Of the 20 grade II astrocytic tumors analysed with this intragenic marker (D4S1295), only 6 samples were informative (heterozygous), of which 3 showed LOH. For grade IV astrocytic tumors, 5 out of 20 samples were informative, of which 2 showed LOH (Fig 3). A cumulative LOH status in both grade II and IV astrocytic tumors was 45.5% (5/11) (Table 2), however a definitive LOH frequency could not be determined due to the small number of informative samples. Hence we tried to use intragenic SNP markers. Of the 10 SNP markers selected for LOH analysis two markers (rs167853 & rs6553016) did not work during standardization. Hence LOH analysis was done with 8 SNP markers. With these SNP markers, an increased number of informative (heterozygous) samples were detected which were suitable for LOH analysis.

**Table 2:** Summary of LOH analysis of *FAT *gene locus in astrocytic tumors (20 grade II and 20 grade IV) with microsatellite markers.

Microsatellite markers D4S		D4S3173	D4S2827	D4S1295	D4S2643	D4S2672
**Informative samples**	**Grade II**	U	U	6/20 (30%)	U	U
	
	**Grade IV**	U	U	5/20 (25%)	U	U

**LOH per informative samples**	**Grade II**	U	U	3/6 (50%)	U	U
	
	**Grade IV**	U	U	2/5 (40%)	U	U

**Total LOH per informative samples** **(Grade II & IV)**		U	U	**5/11 (45.45%)**	U	U

Only one marker (D4S1295) was informative, other 4 markers were uninformative (U).

### Samples

**Figure 4:**
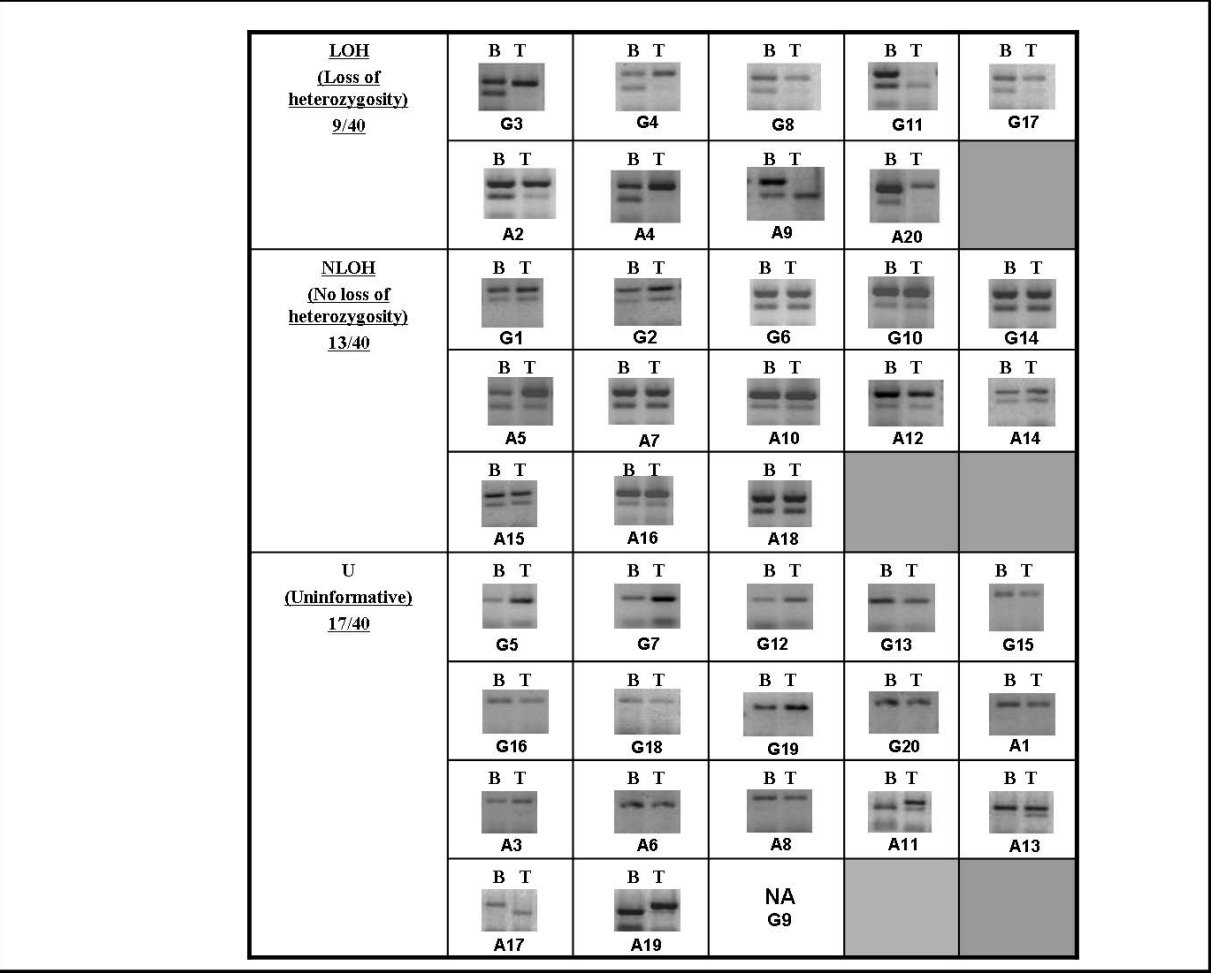
**Representative gel photographs indicating loss of heterozygosity (LOH), no LOH (NLOH) and uninformative (U) results in high (G) and low (A) grade astrocytic tumors (20 each) with one SNP marker (rs450230)**. B indicates normal leucocyte DNA and T indicates tumor DNA. NA is not amplified. All photographs have been cropped and inverted to a white background for clarity.

The LOH analysis with SNP markers was done in 20 grade II and 20 grade IV astrocytic tumors. Out of eight markers used LOH was detected with six markers. A representative LOH profile of all the samples with one marker (SNP marker rs450320) is shown in Fig 4. Using these SNP markers, 38/40 of the samples analyzed were informative. Tumor IDs- A6, A7, A17, G7, G10 and G11 showed LOH with more than one SNP markers studied (Table 3). LOH observed in grade II astrocytic tumors was 42.10% (8/19) and that of grade IV astrocytic tumors was similar, being 57.89% (11/19). Overall LOH frequency with SNP markers observed was 50% (19/38) in all (grades II and IV) the samples studied (Table 4).

**Table 3:** Loss of heterozygosity analysis of the FAT gene with 8 intragenic SNP markers in 20 grade II (A) and 20 grade IV (G) astrocytic tumors.

Low grade Diffuse Astrocytoma (Grade II)																				
**SNP markers**	**A1**	**A2**	**A3**	**A4**	**A5**	**A6**	**A7**	**A8**	**A9**	**A10**	**A11**	**A12**	**A13**	**A14**	**A15**	**A16**	**A17**	**A18**	**A19**	**A20**

**rs2276930**	U	U	U	U	U	U	U	U	U	U	U	U	U	U	U	U	U	U	U	U

**rs3733414**	**NL**	**NL**	U	U	U	**L**	**L**	**NL**	**NL**	**NL**	**NL**	**NL**	**NL**	**NL**	**NL**	**NL**	U	**NL**	**NL**	U

**rs455600**	U	U	U	U	U	**L**	U	**NL**	**NL**	U	**NL**	U	**NL**	U	U	U	**L**	**NL**	U	U

**rs213090**	**NL**	**NL**	U	U	**NL**	U	**L**	U	**NL**	U	**NL**	U	U	U	U	U	**L**	**NL**	**L**	U

**rs450320**	U	**L**	U	**L**	**NL**	U	**NL**	U	**L**	**NL**	U	**NL**	U	**NL**	**NL**	**NL**	U	**NL**	U	**L**

**rs7663350**	**NL**	**NL**	U	U	**NL**	**NL**	U	U	U	U	U	**NL**	U	U	U	**NL**	U	U	U	**NL**

**rs1193110**	U	**NL**	U	U	U	U	U	U	U	U	U	U	U	U	U	U	U	U	**NL**	U

**rs1298865**	U	U	U	U	U	U	U	U	U	U	U	U	U	U	U	U	U	U	U	U

**Glioblastoma multiforme (Grade IV)**																				

**SNP markers**	**G1**	**G2**	**G3**	**G4**	**G5**	**G6**	**G7**	**G8**	**G9**	**G10**	**G11**	**G12**	**G13**	**G14**	**G15**	**G16**	**G17**	**G18**	**G19**	**G20**

**rs2276930**	U	U	U	U	U	U	U	U	U	U	U	U	U	U	U	U	U	U	U	U

**rs3733414**	U	U	U	U	**NL**	U	**L**	U	U	**L**	**L**	U	U	**NL**	**NL**	U	U	U	**NL**	**NL**

**rs455600**	**NL**	U	**NL**	U	U	U	**L**	U	**L**	U	**NA**	**NL**	U	U	U	U	U	U	U	**NL**

**rs213090**	**L**	**L**	U	U	U	U	U	**NL**	U	U	**NL**	U	**NL**	U	U	U	U	U	U	**L**

**rs450320**	**NL**	**NL**	**L**	**L**	U	**NL**	U	**L**	**NA**	**NL**	**L**	U	U	**NL**	U	U	**L**	U	U	U

**rs7663350**	U	U	**NL**	U	U	**NL**	U	**U**	**NL**	U	U	U	U	U	U	U	U	**NL**	**NL**	**NL**

**rs1193110**	U	U	U	U	U	U	U	U	U	**L**	U	**NL**	U	U	U	U	U	U	U	U

**rs1298865**	U	U	U	U	U	U	U	U	U	U	U	**L**	U	U	U	U	U	U	U	U

L is loss of heterozygosity, NL is no loss of heterozygosity, U is uninformative and NA is not analysed. Loss of heterozygosity was detected in 42% of grade II and 58% of grade IV astrocytic tumors.

**Table 4:** Summary of LOH analysis of *FAT *gene locus in astrocytic tumors (20 grade II and 20 grade IV) by RFLP using SNP markers.

Summary of LOH detected with each SNP markers									
**SNP-Markers**		**rs22 76930**	**rs37 33414**	**rs45 5600**	**rs21 3090**	**rs45 0320**	**rs76 63350**	**rs11 93110**	**rs12 98865**

**Informative samples**	**Grade II**	0/20 (0%)	15/20 (75%)	7/20 (35%)	9/20 (45%)	12/20 (60%)	7/20 (35%)	2/20 (10%)	0/20 (0%)
	
	**Grade IV**	0/20 (0%)	8/20 (40%)	6/20 (30%)	6/20 (30%)	10/20 (50%)	0/20 (0%)	2/20 (10%)	1/20 (5%)

**LOH per informative samples**	**Grade II**	0/20 (0%)	2/15 (13.33%)	2/7 (28.57%)	3/9 (33.33%)	4/12 (33.33%)	0/7 (0%)	0/2 (0%)	0/0 (0%)
	
	**Grade IV**	0/20 (0%)	3/8 (37.5%)	2/6 (33.33%)	3/6 (50%)	5/10 (50%)	0/0 (0%)	1/2 (50%)	1/1 (100%)

**Total LOH** **(Grade II & IV)**		**0/20 (0%)**	**5/23** **21.73%**	**4/13** **30.73%**	**6/15** **40%**	**9/22** **40.90%**	**0/7** **(0%)**	**1/4** **25%**	**1/1 100%**

									
**LOH per informative samples**									

**Samples**		**Grade II (N = 20)**		**Grade IV (N = 20)**			**Grade II&IV (N = 40)**		

**Overall SNP LOH per** **informative samples**		8/19* (42.10%)		11/19* (57.89%)			**19/38** **(50%)**		

And SNP LOH detected per informative samples (Grade II and IV) with all SNP markers.

* In both grade II and grade IV one sample each were uninformative with all the SNP markers used.

### *FAT *expression study by RT-PCR

**Figure 5:**
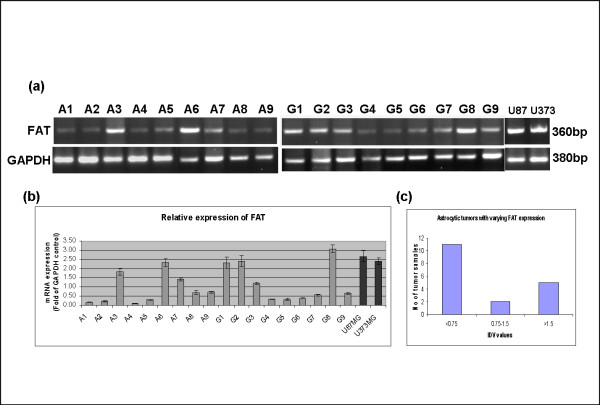
**(a) RT-PCR of the mRNA transcript for FAT and GAPDH (used as internal control) in astrocytic tumors (A = grade II and G = grade IV)**. (b) Densitometric analysis of the *FAT *mRNA expression, normalized with GAPDH. The bars in black indicate U87MG and U373MG, delineating high expression of FAT mRNA. (c) On the basis of IDV (<0.75, 0.75–1.5 and >1.5) tumors were grouped into 3 groups. Most of the tumors could be clustered in two groups, one of which has comparatively lower FAT expression then the other group, the difference was statistically significant (p = 0.000001 by Students t Test).

*FAT *expression was determined by means of reverse transcriptase (RT)-PCR in 18 primary astrocytic tumors (9 grade II and 9 grade IV and two glioma cell lines (U87MG and U373MG)). RT-PCR was done in duplicates. Densitometric analysis was done for the FAT mRNA expression, normalized with GAPDH. There was no significant difference in the expression pattern of *FAT *with the grading of the tumors (Fig 5). On the basis of IDV, tumors were divided into three groups (with normalized integrated density value (IDV) of <0.75, 0.75–1.5 and >1.5) (Fig 5C). Most of the tumors were either with IDV of less than 0.75 (11/18) or greater than 1.5 (5/18) with very few (2/18) in the intermediate cluster (IDV 0.75–1.5). The group with IDV <.75 has been taken as the low expressor group, the one with >1.5 has been taken as high expressor, while the group from 0.75 to 1.5 was indeterminate and has not been taken into account in the statistical comparison. The mean normalized IDV for low expressing group (IDV<0.75) was 0.41 (SD = 0.22) and that of high expressing group (IDV>1.5) was 2.18 (SD = 0.59). When we compared the high and low expressors group, the difference was statistically significant (p = 0.000001 by Students t Test). However, both the glioma cell lines showed high expression (IDV more than 1.5) of *FAT *mRNA.

## Discussion

In this study we have used RAPD-PCR technique, a locus non-selective DNA fingerprinting technique, to identify alteration(s) in the astrocytic tumors of WHO grade II and IV, taking peripheral blood leucocyte DNA of the same patient as a normal control. With RAPD primer 80/07 a loss of a band (500 bp) was detected in 33% (4/12) of the grade II astrocytic tumors studied. The high frequency of this alteration indicated that this alteration was a feature associated with tumorigenesis in a significant proportion of these tumors. Further characterization of the corresponding band in normal DNA by Southern hybridization, cloning, sequencing followed by homology search in the public domain genome database using BLAST search tool showed 100% homology to the *FAT *at exon2-intron2 junction on chromosome 4q34-q35 locus. It is a novel finding as alteration of the *FAT *gene in astrocytic tumors is not reported in literature till date.

RAPD-PCR with multiple primers scans the whole genome in an unbiased manner for any locus and has the potential of detecting genomic regions that are altered and as yet unidentified in astrocytic tumors. The RAPD primers which are short 10-mer random nucleotide sequence acts both as forward and reverse primer. This single primer binds to sites on both the complementary strands of the genomic DNA and priming occurs depending on the sequence match with the template DNA. The best of the primer binding sites on the template often match only about 6–8 bases out of 10 bases at the 3 'end of the primer[18]. Amplification takes place from those regions of the genome where the primer binds in correct orientation and the binding sites are within an amplifiable distance of each other and it requires small amount of DNA (50–100 ng of DNA per PCR). This is important because of the possibility of shearing and low retrieval after extracting tumor DNA from cryostat sections.

The DNA most easily amplified usually constitutes shorter (500 bp-2 kb), multiple products that could easily be resolved on agarose gel. Scoring of the alterations [loss/gain/change in the intensity of band(s)] can be done by simply comparing the banding pattern of the tumor DNA with the normal DNA. The fact that it is not restricted to a defined locus enables its use to hunt for genomic regions that are not identifiable by published literature or not immediately obvious after analysis of sequences. The possibility of obtaining non-reproducible results in RAPD analysis has been discussed in published literature[19]. However, this is not insurmountable and RAPD results are reproducible, as evident from the available literature using this technique[10,11,14,20-22]. In this work all the required precautions[23] have been taken to maintain the reproducibility of the results. Altered bands were checked at least thrice for reproducibility and each alteration was scored by two independent observers. To avoid false positives, we compared the intensities of the preceding and succeeding bands of the altered band in both the normal and tumor DNA while scoring the alteration in the tumor.

After localization of the altered RAPD fragment to the *FAT *gene locus, and no changes being observed in the sequence of the primer binding regions, further analysis was done using locus specific markers of microsatellite and SNP-RFLP markers to determine the LOH status in the tumors. Frequent LOH of a specific chromosomal region is considered to be an indicator of a loss of a closely linked tumor suppressor gene. LOH of the *FAT *locus was detected in 48% (5/11) of the informative samples with the intragenic (D4S1295) marker. Four markers flanking the gene were uninformative (homozygous). Using 8 intragenic SNP markers, 38 out of 40 samples initially taken for LOH analysis were found to be informative and LOH was detected in 50% (19/38) of the informative samples. To the best of our knowledge till date there is no literature available directly linking the *FAT *locus to glial tumorigenesis. SNP analyses in gliomas have been performed by many researchers on other specific chromosomal loci, using SSCP and PCR-RFLP methods and LOH status was correlated to tumor stage and progression[24,25]. In our study, the use of SNP-RFLP has led to a better detection of LOH in both grade II and IV astrocytomas. The similar frequency of LOH of this locus in both low and high grade tumors indicates that this locus is probably affected early in tumorigenesis.

LOH and/or deletion of the chromosome 4q34-35 region (which harbors *FAT *gene) using microsatellite markers was earlier found in grade IV Glioblastoma Multiforme (GBM)[26], though the gene itself was never implicated. Many other tumors like Small Cell Lung Carcinoma[27], hepatocellular carcinoma[28] and cervical carcinoma[29] etc showed alterations in this chromosomal region. Similarly CGH analysis also showed loss of Chromosome 4q34-q35 locus in hepatocellular carcinoma[30] and oral cancer[31]. In all these LOH studies, a significant association of 4q34-35 region with increased risk of progression to higher grade or with the malignancy of the tumors was suggested. Since the *FAT *gene is located in this region it may have an important role to play in the development and progression of various tumors including astrocytic tumors. Our results, showing deletion of *FAT *locus identified with RAPD technique followed by detection of frequent LOH of the locus using microsatellite and intragenic SNP markers, further strengthen the possibility of association of the locus in the development or progression of the astrocytic tumors.

Expression of *FAT *was checked in a panel of 18 astrocytic tumors (9 Grade II and 9 Grade IV), On the basis of IDV, we could cluster most of the tumors in two groups, one of which has comparatively lower FAT expression then the other group. However the two glioma cell lines showed high expression of *FAT*. There is no literature n the pattern of FAT expression in normal human brain. Also not much is known about FAT regulation in primary glial tumors and cell lines. How *FAT *is regulated in cell lines and whether FAT expression in cell lines is a true reflection of the satus in primary tumors is again not clear and needs to be studied.

*FAT *is a human orthologue of *Drosophila *tumor suppressor gene *fat *and is a member of the cadherin gene family. The *fat *gene in Drosophila is essential for controlling cell proliferation during its development. Disruption of *fat *causes imaginal disc tumors in Drosophila[32]. The *fat *gene was first discovered in *Drosophila *followed by the identification of its orthologue in man[33], rat[34], mouse[35] and zebrafish[36]. In humans, the tissue distribution of *FAT *transcripts in fetal and adult tissues has been analyzed in detail by Dunne *et al*[33] who showed that *FAT *mRNA is present in many epithelial and some endothelial and smooth muscle cells. In human fetal tissues, high levels of *FAT *transcripts were found in kidney, lungs, and eye epithelia, which were down regulated in the corresponding adult tissues. Expression analysis in mouse [35] revealed mFAT expression early in pre-implantation stage (eight cell stage). *In situ *expression analyses showed wide spread expression throughout post-implantation development, notably in the limb buds, bronchial arches, and in the proliferating ventricular zones in the brain. RT-PCR analysis of mFAT in adult mouse tissues detected widespread expression in many tissues and cell types. They suggested the role of *FAT *in cell proliferation and differentiation in mouse. *FAT *also serves as a proximal element of signaling pathways necessary for cell migration [37,38]. *FAT *tumor suppressor gene is an upstream regulator of the Salvador-Wart-Hippo (SWH) signaling pathway [39,40] which is well established in Drosophila. This pathway is conserved in human but the role of *FAT *in human cancer is not clear and not much literature is available about *FAT *and its effect on SWH pathway in humans. The downstream effector molecule of SWH pathway is YAP. Like TGFβ, in mammal, SWH pathway via YAP is found to have both oncogenic [41-43] and tumor suppressor effects [44]. *FAT *might have an important role in oncogenesis in humans. Recently, Nakaya et al[31] has shown homozygous deletion of *FAT *gene in oral cancer using CGH-array and low mRNA expression of *FAT *gene suggested importance of *FAT *in the development of oral cancer. Role of *FAT *gene is also suggested in development of fallopian tube cancer in a patient of MRKH (Mayer Rokitansky Kuster Hauser) syndrome [45]. Based on the signaling pathways linked with FAT, with several of the signaling pathways associated with Drosophila ortholgues have been worked out in humans, a comparative study of signaling pathways and other properties of the two tumor groups (high and low expressing) would be useful and is planned for the future.

## Conclusion

Our results show frequent LOH at the *FAT *locus in primary human glial tumors. Based on FAT expression, these tumors may be divided into two groups showing low and high expression respectively. Our results also show that RAPD analysis is a reliable method in scanning the genome for identification of novel genomic alterations in tumors. Not only can it address the issues related to the overall extent and nature of genomic instability, but specific regions affected in a particular tumor type can also be identified. To further understand the functional role of *FAT *gene in tumorigenesis, we are in the process of analyzing its expression and methylation status in primary astrocytic tumors and in cell lines. We are also trying to determine whether the low and high expressor groups have any differences with regard to altered signaling pathways (especially the SWH pathway) and in the tumor phenotype.

## Abbreviations

RAPD: Random Amplification of Polymorphic DNA; DA: Low grade diffuse astrocytoma; AA: Anaplastic astrocytoma; GBM: Glioblastoma Multiforme; LOH: Loss of heterozygosity; SNP: Single Nucleotide Polymorphism; RFLP: Restriction fragment length polymorphism; RT-PCR: Reverse transcriptase polymerase chain reaction; TSG: tumor suppressor gene; GAPDH: Glyceraldehyde: 3: phosphate dehydrogenase.

## Competing interests

Authors have no personal or financial conflict of interest and have not entered into any agreement that could interfere with the processing of paper for publication.

## Authors' contributions

KC carried out the samples processing, PCR standardization, RAPD analyses, cloning, sequence alignment, LOH experiments & analysis and drafted the manuscript. AM participated in the sample processing and RAPD analysis. SP participated in RNA sample processing and helped write the manuscript. TS participated in microsatellite LOH experiments and helped write the manuscript. PC participated in sequence alignment, statistical analysis and helped write the manuscript. CS carried out the pathological analysis of the tumor samples, including determining the proportion of tumor and normal cells and helped write the manuscript. AKM carried out the tumor samples selection and analysis and helped write the manuscript. SS conceived the study, helped in study design, statistical analysis, coordination, and helped write the manuscript.

All the authors have read and approved the final version of the manuscript.

## Pre-publication history

The pre-publication history for this paper can be accessed here:

http://www.biomedcentral.com/1471-2407/9/5/prepub
